# Left-Colonic Intussusception Due to Necrotic Lipoma in a Young Male Patient

**DOI:** 10.14309/crj.0000000000001852

**Published:** 2025-10-03

**Authors:** Kimberly Ho, Leon Zhao, Yousef Elfanagely, Breton Roussel

**Affiliations:** 1 Brown University Health Department of Medicine, Providence, RI; 2 The Warren Alpert Medical School of Brown University, Providence, RI; 3 Mount Sinai, Department of Gastroenterology, Brown University Health Department of Gastroenterology, Providence, RI; 4 University Gastroenterology, Providence, RI

**Keywords:** intussusception, lipoma, colon

## Abstract

We present a rare case of left-colonic intussusception in a 31-year-old patient caused by a large, necrotic, ulcerated lipoma. This case is noteworthy as the patient is a young man, which is an atypical population for this presentation. The left colon is also a relatively uncommon site for colonic lipomas. Overlying colonic mucosal ischemia and large size of the lipoma precluded endoscopic treatment and ultimately required a partial colectomy.

## INTRODUCTION

Colo-colonic intussusception is an invagination of a proximal bowel segment into the lumen of a distal bowel segment. This process is rare among adult patients with an incidence of 2–3 cases per million annually in the general population.^1^ Between 60% and 70% of intussusceptions seen in adult patients are caused by malignant carcinomas.^2^ Lipomas, on the other hand, are uncommon and benign causes of colonic intussusception. A lipoma is a tumor of nonepithelial benign adipose tissue that occurs in the gastrointestinal tract and can serve as a lead point to produce invagination within the lumen to cause intussusception. We present a patient with colo-colonic intussusception in the setting of a left colon lipoma.

## CASE REPORT

A 31-year-old man without any past medical history presented to the emergency department with 3 weeks of constant abdominal pain localized to the left lower quadrant. The pain was nonradiating, rated 7 out of 10 in severity, worsened with eating and did not improve with acetaminophen use. The patient also reported nausea and vomiting after meals and noted blood in stool for the past 4 days with every bowel movement. On the day of admission, he had a bowel movement and was able to pass flatus. The patient denied any previous abdominal surgeries and had no family history of colon cancer. He denied history of alcohol, tobacco, or recreational drug use.

**Figure 1.**
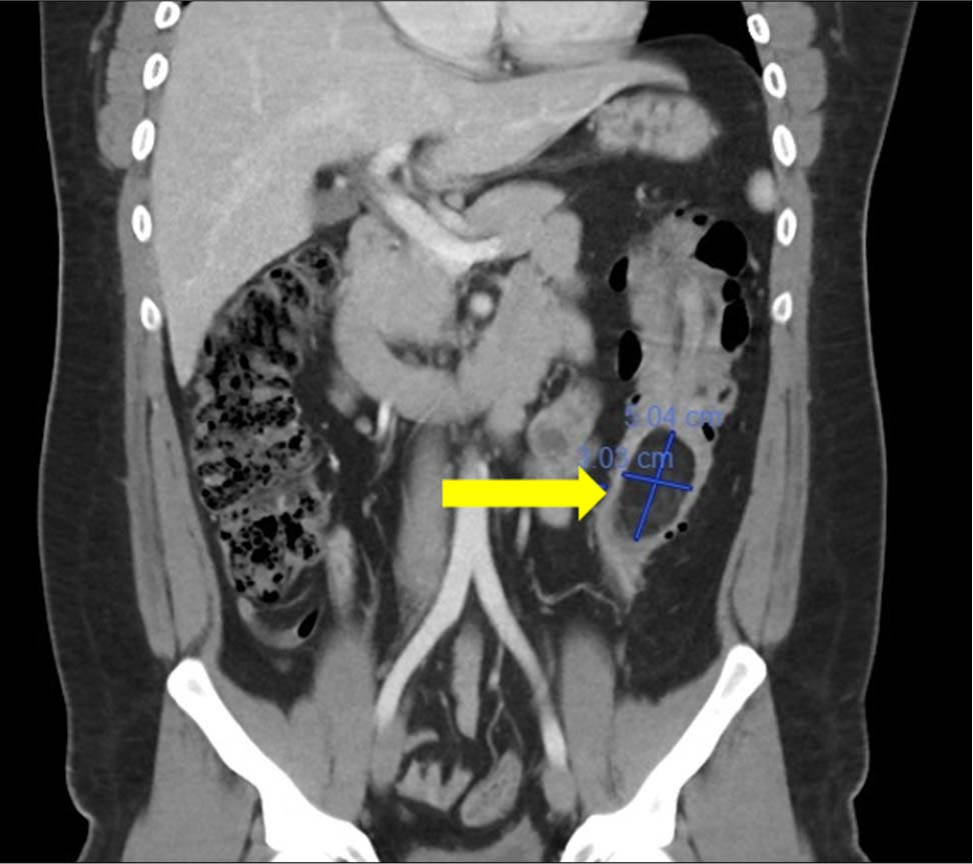
Contrast enhanced CT, coronal view showing intussuscepting colonic lipoma in the descending colon (yellow arrow). CT, computed tomography.

The patient was hemodynamically stable on admission. Initial laboratory tests revealed a mild leukocytosis but were otherwise within normal limits, including hemoglobin and hematocrit. On physical examination, the abdomen was soft and mildly distended with tenderness with light and deep palpation on the left side of his abdomen, most prominently in the left lower quadrant. There were no peritoneal examination findings. An initial abdominal contrast-enhanced computed tomography scan was performed, which revealed a 3.5 × 3.7 × 5.5 cm descending colon fat density mass with intussusception of the splenic flexure into the proximal descending colon for a length of approximately 14 cm (Figure 1). No abnormal proximal bowel dilatation was seen to suggest obstruction, and there were no signs of bowel ischemia. The appendix was also normal.

**Figure 2.**
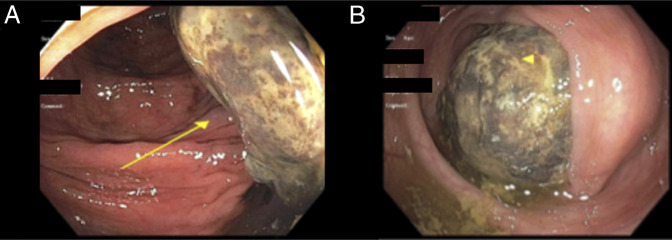
Imaging from colonoscopy depicting partially obstructing, ulcerated, necrotic submucosal mass serving as lead point for left colonic intussusception. (A) Mass at splenic flexure. (B) Partially obstructing same submucosal mass.

The patient was admitted to the colorectal surgical service, and the gastroenterology service was paged 7 days later from their service for an urgent endoscopic evaluation. Colonoscopy revealed a 6 cm submucosal mass with overlying ischemic and ulcerated mucosa in the proximal descending colon. This examination was consistent with localized area of left colonic intussusception with a large submucosal mass as lead point. The protruding mucosa was partially obstructing but was able to be traversed with a pediatric colonoscope (Figure 2). There was no bleeding from the lesion. 5 mL India ink 2× was successfully injected for tattooing at the base of the intussusception distal to the lesion to assist with subsequent surgical localization. Colonoscopic examination and insufflation with carbon dioxide failed to reduce the intussusception. No biopsies were performed on the mass during endoscopic evaluation given concern for increased risk of perforation in the setting of severe mucosal ischemia and ultimate plan for surgical resection.

Following the colonoscopy, the patient underwent a laparoscopic partial colectomy and splenic flexure mobilization. No images from the surgery were taken. The final pathology report showed a gross dissection of bowel intussusception, associated with the large, colonic, submucosal lipoma. There was fat necrosis, and the overlying colonic mucosa had ischemia, ulceration, and necrosis, namely features of ischemic injury with ulceration and hemorrhage. There was no evidence of malignancy. The patient recovered well after surgery and was discharged shortly thereafter without complication.

## DISCUSSION

Colonic lipomas were first described by Bauer in 1757.^3^ There are 190 case reports since the early 1900s in our literature search of intussusception due to colonic lipomas. To date, there are 20 case reports of intussusception seen specifically due to left sided or descending colonic lipomas. Most cases of colonic intussusception due to a colonic lipoma are observed in female patients.

There were several unique points about this case that we would like to highlight. First, our patient is a young man, which is not within the typical demographic for this presentation. He also presented with a large, necrotic, ulcerated lipoma in the left colon, which is an uncommon location for presentation. The lipoma appeared submucosal, sessile, and was large (5.5 cm), which ultimately required partial colectomy.

Lipomas arise more frequently in middle-aged adults in their fifth decade with a slight female predominance.^4^ The main clinical symptom associated with intussusception from colonic lipoma is abdominal pain, which is present in up to 83% of cases in literature.^5^ There may also be blood (16%), mucous with blood (4%), or losses from the rectum and hematochezia (11%).^5^ Typically, lipomas larger than 5 cm will cause symptoms, including nonspecific abdominal pain and bleeding, which was seen in our case.^6^

Most lipomas are found incidentally during routine screening colonoscopy and do not require intervention. Diagnosis of colonic lipomas is typically made by characteristic features during the endoscopic examination including submucosal location, characteristic pillow sign, mucosal tenting, and extruded submucosal fat after mucosal biopsy. Computed tomography scan is also specific for identifying characteristic features of a lipoma. The frequency of lipomas causing intussusception in the colon are generally 38% in the ascending colon, 22% in the transverse colon, 19% in the cecum, 13% in the descending colon, and 8% in the sigmoid.^6^ The cecum is considered the most prone area to intussusception, particularly where the ileum meets the large intestine at the ileocecal junction.^7^ Colonic lipomas are generally sessile in 90% of cases and rarely pedunculated.^8–10^ There is no clear evidence suggesting that the urgency of removal is determined by whether the lipoma is pedunculated or sessile. Instead, the decision for urgent removal, particularly surgical resection, is generally guided by clinical indications and complications, such as intussusception, intestinal obstruction, or when the lipoma exceeds 2 cm in size.^5,6,11^

Typically, large lipomas are generally not amenable to endoscopic resection due to their size, submucosal origins, and overlying ischemic tissue. However, several advanced endoscopic techniques have shown success in managing large lipomas (>8 cm) causing intussusception. These methods include piecemeal endoscopic mucosal resection (EMR), endoscopic unroofing and mucosal resection, endoscopic submucosal dissection (ESD), and endoscopic mucosotomy with snare resection.

Endoscopic unroofing and mucosal resection involves incising the surface of the lipoma and removing the upper portion with a snare, allowing the fatty tissue to extrude. Subsequently, EMR is performed to remove any residual lipoma in fragments using a snare.^12^ This technique was first introduced in 1997 and successfully resected a 5 cm non-necrotic lipoma in Japan in 2003.^13^ Although this approach minimizes the risk of perforation, which is higher with EMR alone, there remains a risk of leaving tumor remnants.^12^

ESD is a more precise technique, allowing for the removal of the lipoma in one piece by dissecting the submucosal layer. A Japanese study found ESD to be a safe and effective method for resecting colorectal lesions ranging from 2 to 8 cm, potentially outperforming EMR.^14^ Another case in Japan successfully performed ESD on an ileal lipoma, emphasizing the advantages of avoiding bulky snares and the postoperative complications associated with surgery.^15^

Endoscopic mucosotomy with snare resection involves incising the mucosa on the surface of the lipoma to expose the fatty tissue, followed by piecemeal resection with a snare. This method has been successfully used to resect an 8 cm colonic lipoma in a patient with recurrent intussusception who refused surgical intervention.^16^

Based on the literature and ACG guidelines, advanced endoscopic techniques are preferred for large colonic lipomas, especially when the mass is benign, accessible, and can be safely removed endoscopically.^17^ Factors favoring endoscopic resection include a pedunculated or well-circumscribed lesion, low suspicion for malignancy, and the absence of ischemia. Notably, intussusception is not an absolute contraindication for endoscopic management, as evidenced by successful cases treating large lesions with intussusception. Endoscopic methods are associated with significantly lower 30-day mortality and fewer major adverse events compared with surgery.^17^

In our case, the contraindication to endoscopic management was the evidence of mucosal ischemia and compromised bowel viability, which necessitated minimally invasive surgical treatment. Necrosis of the lipoma was another unique characteristic of this case. We did not identify any significant risk factors or relevant personal or family medical history that could have contributed. It was most likely necrotic since the mass was located in a watershed area and large, approximately 6 cm in size. Notably, 75% of cases with lipomas larger than 4 cm present with symptoms, though not all larger lipomas are symptomatic.^8^ It is possible that our patient was asymptomatic or only mildly symptomatic for an extended period, with more persistent abdominal pain becoming evident only when the patient developed clear signs of intussusception.

Surgical resection remains the preferred option in cases of failed endoscopic resection, high risk of perforation, suspected malignancy, or deeper tissue involvement. Moreover, some lipomas are associated with severe symptoms and complications. One reported case involved a giant necrotic colonic lipoma causing intussusception, which was visualized on colonoscopy. The patient, who had multiple comorbidities, including previous cerebrovascular accidents on clopidogrel, diabetes, stage 5 chronic kidney disease, and iron deficiency anemia, ultimately required surgical resection due to desaturation in the endoscopy suite.^10^

In summary, endoscopy and colonoscopy remain a critical diagnostic tool for evaluating these masses. Direct visualization can aid in characterization, with findings such as the endoscopic pillow sign often associated with benign lipomas.^11^In addition, biopsies can provide histopathological insights, including the naked fat sign, where fat visibly spills out of the lesion upon biopsy.^11^ Laparoscopic surgery is also the optimal treatment of choice generally for large lipomas.^2^ Specifically, surgical resection should be performed if the lipoma is larger than 4 cm in diameter, the preoperative diagnosis is suspicious of malignancy, the lesion is symptomatic or causing intussusception, there is wall infiltration, and the lipomas cannot be completely removed endoscopically.^18^

## DISCLOSURES

Author contributions: K. Ho drafted the manuscript, and is the article guarantor. L. Zhao, Y. Elfanagely, and B. Roussel critically revised the manuscript.

Financial disclosure: B. Roussel: consultant for Phathom Pharmaceutics.

Informed consent was obtained for this case report.

## ABBREVIATIONS:

ACG: American College of Gastroenterology
EMR: endoscopic mucosal resection
ESD: endoscopic submucosal dissection
